# Advanced nanoparticles that can target therapy and reverse drug resistance may be the dawn of leukemia treatment: A bibliometrics study

**DOI:** 10.3389/fbioe.2022.1027868

**Published:** 2022-10-10

**Authors:** Rui Wang, Changming Zhao, Shuxia Jiang, Zhaohua Zhang, Chunmei Ban, Guiping Zheng, Yan Hou, Bingjin Jin, Yannan Shi, Xin Wu, Qiangqiang Zhao

**Affiliations:** ^1^ Department of Hematology, Shandong Second Provincial General Hospital, Jinan, China; ^2^ Department of Hematology, The Qinghai Provincial People’s Hospital, Xining, China; ^3^ Department of Hematology, Hematology Department, The People’s Hospital of Liuzhou City, Liuzhou, China; ^4^ Department of Pharmacy, The Qinghai Provincial People’s Hospital, Xining, China; ^5^ Department of General Medicine, Ganmei Hospital, Kunming First People’s Hospital, Kunming, China; ^6^ Department of Spine Surgery, Third Xiangya Hospital, Central South University, Changsha, China

**Keywords:** nanoparticles, target therapy, drug resistance, leukemia, chemotherapy

## Abstract

With the development of nanomedicine, more and more nanoparticles are used in the diagnosis and treatment of leukemia. This study aimed to identify author, country, institutional, and journal collaborations and their impacts, assess the knowledge base, identify existing trends, and uncover emerging topics related to leukemia research. 1825 Articles and reviews were obtained from the WoSCC and analyzed by Citespace and Vosviewer. INTERNATIONAL JOURNAL OF NANOMEDICINE is the journal with the highest output. The contribution of FRONTIERS IN BIOENGINEERING AND BIOTECHNOLOGY is also noteworthy. The three main aspects of research in Nanoparticles-leukemia-related fields included nanoparticles for the diagnosis and treatment of leukemia, related to the type and treatment of leukemia, the specific molecular mechanism, and existing problems of the application of nanoparticles in leukemia. In the future, synthesize nano-drugs that have targeted therapy and chemotherapy resistance according to the mechanism, which may be the dawn of the solution to leukemia. This study offers a comprehensive overview of the Nanoparticles-leukemia-related field using bibliometrics and visual methods for the first time, providing a valuable reference for researchers interested in Nanoparticles-leukemia.

## 1 Introduction

Cancer has always been a thorn in the hearts of human beings worldwide, and leukemia is even more worrying for researchers because of its high incidence and low survival rate (Wu et al., 2021a; Wu et al., 2021b). Leukemia is a highly invasive hematological tumor with complex genetic and biological characteristics (Du et al., 2021; Jiang et al., 2022; Wu et al., 2022). There are a variety of treatments for leukemia, including chemotherapy, radiotherapy, stem cell transplantation, immunotherapy, targeted therapy, and so on (Kaldor et al., 1990; Montserrat and Dreger, 2015; Woyach, 2019; Locatelli et al., 2021; Lussana et al., 2021). Although immunotherapy such as Autologous chimeric antigen receptor (CAR) T cells has attracted wide attention from researchers recently, chemotherapy has always been the main treatment for leukemia because of its classic and effectiveness. Chemotherapy is one of the primary means to treat leukemia. However, the clinical application still has the following problems: 1. Chemotherapeutic drugs kill leukemic and normal cells and produce adverse effects, including malnutrition, cancer, and chronic pain. 2. The emergence of drug resistance leads to a significant decline in chemotherapy efficacy and a poor prognosis (Lokody, 2014). How to solve the above problems has become an urgent problem to be solved in this field.

Nanotechnology is widely used in medicine, such as tissue repair, diagnosis, and treatment of diseases (Lin et al., 2020a; Lin et al., 2020b; Zhang et al., 2020; Bai et al., 2021; Chen et al., 2021; Zhao et al., 2022). Professor Feng’s team developed a GSH bioimprinted nanocomposite loaded with an FTO inhibitor (GNPIPP12MA). The GNPIPP12MA can not only achieve an anti-leukemia effect by inducing ferroptosis but also enhance the blocking effect of PD-L1 by increasing the infiltration of cytotoxic T cells (Cao et al., 2022). Professor Zhao’s team constructs a novel amino acid nanomedicine for the treatment of T-cell acute lymphoblastic leukemia by combining leukemia cell targeting and immune surveillance stimulation (Li et al., 2022). Nanoparticles can not only treat leukemia by transporting small molecular drugs, antibody drugs, and genes but also carry out photodynamic therapy for leukemia through photosensitizers (Shen et al., 2020). Because the physiological structure of a tumor is different from that of normal tissue, nanoparticles can be passively targeted to the tumor by the Enhanced Permeability and Retention Effect (EPR). EPR refers to the phenomenon that some macromolecular substances of a specific size (such as liposomes, nanoparticles, and some macromolecular drugs) are more likely to penetrate into tumor tissue and remain for a long time (compared with normal tissue). At the same time, nanoparticles can also be modified by the cell membrane, ligand, or aptamer to achieve active targeting (Tatar et al., 2016; Tee et al., 2019). However, the research on nano-drugs that can achieve specific targeting and reverse leukemia drug resistance is still in its infancy.

There are many ways to systematically review a research field, among which the bibliometric method is one of the most commonly used approaches. Bibliometric analysis can study the contributions and collaboration of researchers, organizations, nations, and journals qualitatively and quantitatively and assess academic research’s developmental status and new tendencies (Zhang et al., 2021). The bibliometric method can also consider conventional reviews, meta-analysis, or experimental studies, whose analysis cannot be performed using other approaches (Petersen et al., 2016; Björnmalm et al., 2017; Izci et al., 2021). Based on these advantages, this method is increasingly used to assess academic tendencies and develop guidelines. Hence, we used bibliometric analysis for evaluating and summarizing Nanoparticles-leukemia studies.

This study aimed to objectively delineate the knowledge field and new trends in Nanoparticles-leukemia research from the following four dimensions using two standard bibliometric tools, CiteSpace and VOSviewer. 1) We intended to quantify and identify general information in Nanoparticles-leukemia studies by studying yearly articles, journals, co-cited journals, nations, organizations, researchers, and co-cited researchers. 2) We strived to identify and study the top 100 most cited articles through co-citation reference analyses to assess the knowledge base of Nanoparticles-leukemia. 3) We aimed to identify the knowledge structure and hotspot evolution through keyword and co-citation reference burst analyses (Jiang et al., 2022). Meanwhile, under the analyses of the journals, countries, and keywords of the former top 100 articles and co-cited journals, combined with the analysis content of 3), the research content and possible new directions in the field of Nanoparticles-leukemia were further determined.

## 2 Materials and methods

### 2.1 Study design

**FIGURE 1:**
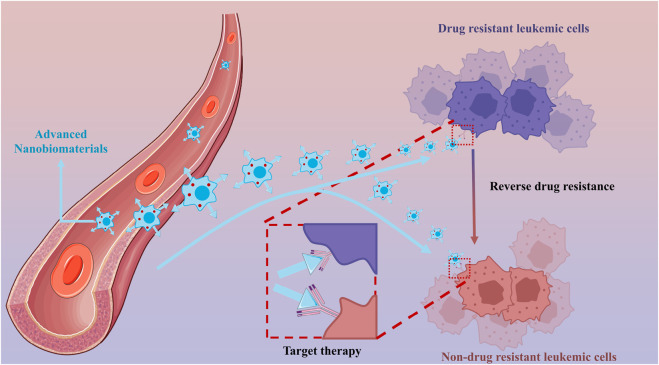
Advanced nanobiomaterials that can target therapy and reverse drug resistance.

This study used bibliometric analysis to study journal papers in Nanoparticles-leukemia. The study is divided into two parts: the first is the study of all the published articles in the Nanoparticles-leukemia field using WOSCC; the second is the analysis of the first 100 highly cited papers in the Nanoparticles-leukemia (Figure 1).

### 2.2 Eligibility criteria

Inclusion criteria included (Wu et al., 2021a) Basic scientific and clinical studies related to nanoparticles-leukemia (Wu et al., 2021b); Studies on diagnosis, treatment, prognosis or prevention related to nanoparticle-leukemia; and (Wu et al., 2022) Original articles and reviews that are closely related to nanoparticles-leukemia. Exclusion criteria were (Wu et al., 2021a) Contents that are not related to nanoparticles-leukemia.

### 2.3 Data collection

Our team used the WoSCC database as it can offer comprehensive data required by the bibliometric software and is considered the most potent database. Data were acquired from the WoSCC database on 2 August 2022, and the relevant data can be found in Supplementary Materials 1. Our main search terms were “Nanoparticles” and “leukemia” (Lin et al., 2020c; Huang et al., 2022). For specific search terms, please refer to Supplementary Material 2. Part I: All articles relevant to the Nanoparticles-leukemia field were downloaded from WoSCC (Time span from the establishment of WoSCC to 2 August 2022). Part II: Two independent researchers manually screened the top 100 most-cited articles relevant to the Nanoparticles-leukemia field. Search conditions: The language was limited to English, and the type of article was restricted to articles and reviews. The retrieval outcomes were downloaded from the recorded contents of “Full Record and Cited References.” (Zhang et al., 2021).

### 2.4 Data analysis and visualization

The most frequently utilized bibliometric programs are VOSViewer, CiteSpace, SCI2, NetDraw, and HistCite. There is no consensus on the best bibliographic approach. After careful consideration, this study utilized VOSviewer and CiteSpace.

Our team used VOSviewer1.6.15 to determine influential journals, co-cited journals, researchers, co-cited researchers, and associated knowledge graphs based on bibliographic information. Moreover, our team created keyword co-occurrence and clustering diagrams based on text data. First, the data were cleaned. For example, “nanoparticle” and “NPs” were unified to “nanoparticles” in the keyword analysis. The project’s other thresholds (T) were set according to different circumstances and marked in the relevant tables and illustrations.

CiteSpace, proposed by Professor Chen Chaomei, is a bibliometric and visualization analysis tool suitable for investigating collaboration, key points, inner architectures, and latent tendencies in a specific field. Hence, our team used CiteSpace6.1.R2 to study and visualize the co-occurrence of nations and organizations, dual maps, high-frequency keyword trends, co-citation references, and citation bursts. The data were cleaned before the investigation; For example, articles from Taiwan were classified as China in the country analyses. The CiteSpace settings are stated below: time span, 1999–2022; years per slice, 1, pruning, minimum spanning tree and pruning sliced networks; selection standards, Top N = 50; and others followed the default.

Microsoft Office Excel 2022 was used to process the annual database of articles. Furthermore, the 2021 journal IF and JCR Division was obtained from the Web of Science in Cites Journal Citation Reports on 11 August 2022.

## 3 Results

### 3.1 The annual growth trend and the number of annual cited departments

**FIGURE 2:**
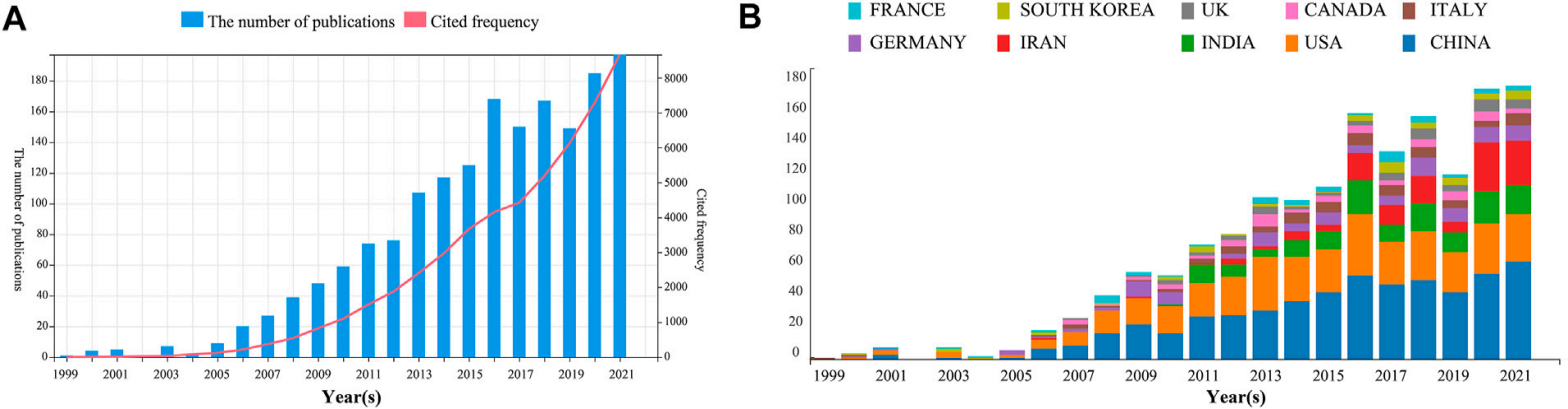
**(A)** Annual publication number and citation frequency of Nanoparticles-leukemia-related studies. **(B)** Composition ratio of the top 10 countries by the publication number.

As per the data acquisition method, our team obtained 1825 articles upon limiting the types of research to articles and reviews and the language to English. Finally, 1825 eligible articles (Supplementary Material 1) published between 1999 and 2022 were selected. From Figure 2A, we can observe that references related to Nanoparticles-leukemia generally showed an increasing trend. It is worth noting that the citations of published articles in 2017–2021 have increased significantly. In addition, we also analyzed the top 10 countries with published articles (Figure 2B): America was leading in publishing articles until 2007 (1999–2007). Since 2008, China has shown great interest in Nanoparticles-leukemia, surpassing the United States to become the country with the most significant number of articles published.

### 3.2 Journals and co-cited journals

Our team utilized VOSviewer to analyze journals and co-cited journals to find the most influential and vital journals concerning the Nanoparticles-leukemia field. The results revealed that 1825 articles related to this field were published in 595 journals. The INTERNATIONAL JOURNAL OF NANOMEDICINE published the most articles (76, 4.1485%) (Table 1). About the country of the publication, US and United Kingdom accounted for more than half (7/12). Among the top 10 journals, nine were from the Q1 JCR division, and their Impact Factors (IF) exceeded 5 (Table 1). Among the 7,934 commonly cited journals, ten were cited > 1000 times. As presented in Table 2, BLOOD had the most significant number of citations (N = 2,458), followed by JOURNAL OF CONTROLLED RELEASE (N = 2038) and BIOMATERIALS (N = 1845). It is not surprising that the journals with high co-citations are all from the Q1 JCR partition and all have high IF values. Furthermore, consistent with the analysis of high-volume journals, the US and United Kingdom accounted for more than half of the country’s distribution, followed by NETHERLANDS.

**TABLE 1:** The top 10 journals of Nanoparticles-leukemia-related research.

RANK	Journal	N	(%)	IF(2021)	JCR division	Country
1	INTERNATIONAL JOURNAL OF NANOMEDICINE	76	4.14847161572052	7.033	Q1	New Zealand
2	JOURNAL OF CONTROLLED RELEASE	34	1.85589519650655	11.467	Q1	NETHERLANDS
3	MOLECULAR PHARMACEUTICS	33	1.80131004366812	5.364	Q1	United States
4	BIOSENSORS BIOELECTRONICS	32	1.74672489082969	12.545	Q1	United Kingdom
5	RSC ADVANCES	31	1.69213973799127	4.036	Q2	United Kingdom
6	INTERNATIONAL JOURNAL OF PHARMACEUTICS	30	1.63755458515284	6.51	Q1	NETHERLANDS
7	BIOMATERIALS	28	1.52838427947598	15.304	Q1	NETHERLANDS
8	NANOSCALE	26	1.41921397379913	8.307	Q1	United Kingdom
9	ACS NANO	24	1.31004366812227	18.027	Q1	United States
	ANALYTICAL CHEMISTRY	24	1.31004366812227	8.008	Q1	United States
	COLLOIDS AND SURFACES B BIOINTERFACES	24	1.31004366812227	5.999	Q2	NETHERLANDS
10	JOURNAL OF BIOMEDICAL NANOTECHNOLOGY	23	1.25545851528384	3.641	Q3	United States

**TABLE 2:** The top 10 co-cited journals of Nanoparticles-leukemia-related research.

RANK	Co-cited journal	N	IF(2021)	JCR division	Country
1	BLOOD	2458	25.476	Q1	United States
2	JOURNAL OF CONTROLLED RELEASE	2038	11.467	Q1	NETHERLANDS
3	BIOMATERIALS	1845	15.304	Q1	NETHERLANDS
4	CANCER RESEARCH	1715	13.312	Q1	United States
5	PNAS	1524	12.779	Q1	United States
6	ACS NANO	1357	18.027	Q1	United States
7	JOURNAL OF THE AMERICAN CHEMICAL SOCIETY	1248	16.383	Q1	United States
8	NATURE	1123	69.504	Q1	United Kingdom
9	ADVANCED DRUG DELIVERY REVIEWS	1102	17.873	Q1	NETHERLANDS
10	INTERNATIONAL JOURNAL OF NANOMEDICINE	1096	7.033	Q1	New Zealand

**FIGURE 3:**
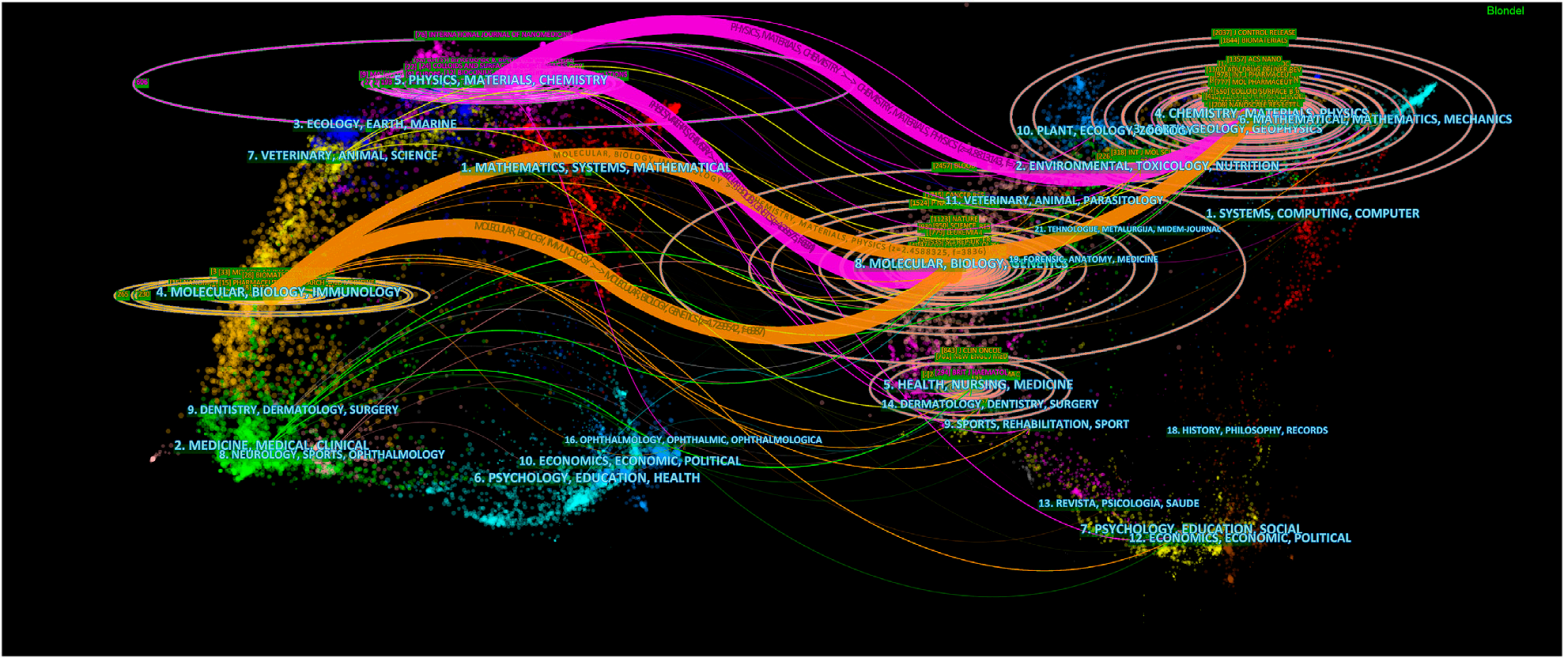
The dual-map overlay of journals related to Nanoparticles-leukemia research. Notes: The citing journals were on the left, the cited journals were on the right, and the colored path represents the citation relationship.

The journal dual-map overlay represents the topic distribution status of journals (Figure 3). Citation journals are on the left, cited journals are on the right, and the color path indicates the citation relationship. Four primary citation path was determined, which meant that research published in Molecular/Biology/Immunology and Physics/Materials/chemistry journals was predominantly cited by a study published in Molecular/Biology/Immunology and Chemistry/Materials/Physics journals.

### 3.3 Country/region and institution

**FIGURE 4:**
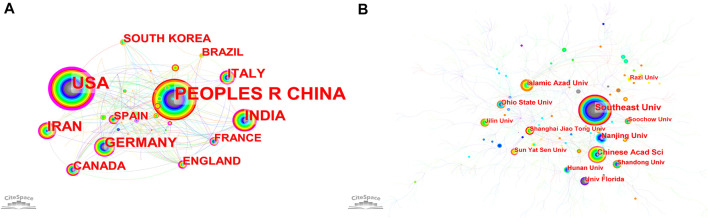
The co-occurrence map of **(A)** countries/regions and **(B)** institutions in Nanoparticles-leukemia research (T ≥ 18). Notes: The node’s size reflects the co-occurrence frequencies, and the links indicate the co-occurrence relationships. The color of the node and line represent different years. Colors vary from grey to orange as time goes from 1999 to 2022; a node with a purple round means high betweenness centrality (>0.1).

A total of 2,174 institutions from 80 countries have published 1825 articles. The most significant number of publications came from China (593, 24.14%), followed by the United States (419, 17.06%) and Japan (161,6.56%) (Table 3). As we have presented in Table 3, the United States, France, and Germany have made indelible contributions in the Nanoparticles-leukemia field (Figure 4A). In addition, according to link color, the United States (1999), Germany (1999), United Kingdom (1999) and China (2000) were the first countries to conduct studies on Nanoparticles-leukemia field. We used minimal spanning tree pruning to clarify the net (Figure 4A). The standard map of unpruned countries contained 80 nodes and 272 links with a density of 0.0861, revealing active collaboration between diverse nations. For example, the United States cooperated with 42 nations, followed by Germany (*n* = 34) and China (*n* = 31).

**TABLE 3:** The top 10 countries/regions and institutions involved in Nanoparticles-leukemia-related research.

RANK	Country/region	N	Percent(%)	Centrality	Institution	N	Percent(%)	Country/region	Centrality
1	PEOPLES R CHINA	593	24.1449511400651	0.03	Southeast Univ	82	2.3768115942029	CHINA	0.1
2	USA	419	17.0602605863192	0.44	Chinese Acad Sci	40	1.15942028985507	CHINA	0.16
3	INDIA	161	6.55537459283388	0.15	Nanjing Univ	34	0.985507246376812	CHINA	0.03
4	IRAN	146	5.94462540716612	0.1	Islamic Azad Univ	29	0.840579710144928	IRAN	0.06
5	GERMANY	108	4.39739413680782	0.18	Ohio State Univ	25	0.72463768115942	United States	0.06
6	ITALY	80	3.25732899022801	0.12	Univ Florida	22	0.63768115942029	United States	0.03
7	CANADA	61	2.48371335504886	0.17	Hunan Univ	20	0.579710144927536	CHINA	0.01
8	SOUTH KOREA	50	2.03583061889251	0.02	Shandong Univ	19	0.550724637681159	CHINA	0.01
9	ENGLAND	49	1.99511400651466	0.12	Sun Yat Sen Univ	18	0.521739130434783	CHINA	0.14
10	FRANCE	45	1.83224755700326	0.19	Shanghai Jiao Tong Univ	18	0.521739130434783	CHINA	0.06
					Jilin Univ	18	0.521739130434783	CHINA	0.02

More than half of the top 10 institutions were from China (6/11), followed by the United States (8/11) (Figure 4B) (Table 3). Southeast Univ (82,2.37%) published the most papers, followed by Chinese Acad Sci, Nanjing Univ, and Islamic Azad Univ (Table 3).

### 3.4 The highly productive author

**FIGURE 5:**
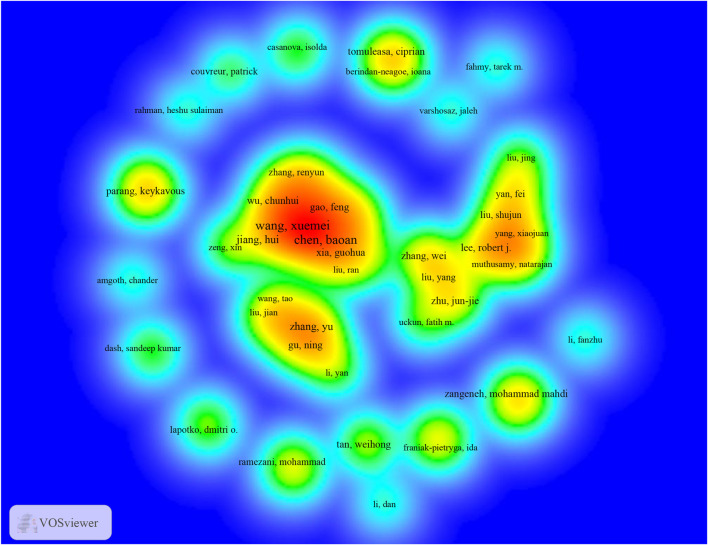
The density map of authors in Nanoparticles-leukemia research (T≥5). Notes: The size of the word, round, and the opacity of yellow are positively related to the publication frequency.

Eight thousand seven hundred forty-one researchers participated in Nanoparticles-leukemia research. Furthermore, 11 published more than 14 papers. Wang, Xuemei wrote the most articles (*n* = 40), followed by Chen, Baoan (*n* = 38), Jiang, hui (*n* = 18) (Table 4). It includes authors who have authored more than five articles (T ≥ 5) (*n* = 80) to construct authors (Figure 5). Such knowledge graphs can present high-frequency researchers. As represented in Figure 5, Wang, Xuemei, Chen, Baoan, Jiang, hui, Gao, Feng and, Xia, guohua were closely linked and formed a group of authors with the reddest color, indicating that the group has made outstanding contributions in the field of Nanoparticles-leukemia. Zhang, Yu, and Gu, Ning constituted the second largest group of authors. Lee, Robert j. and Zhang, Wei constituted the third largest group of authors.

**TABLE 4:** The top 10 authors and co-cited authors of Nanoparticles-leukemia-related research.

RANK	Author	Count	Citation	H-index
1	wang, xuemei	40	1706	64
2	chen, baoan	38	1516	44
3	jiang, hui	18	742	31
4	zhang, yu	18	463	36
5	parang, keykavous	16	342	36
6	gu, ning	15	437	56
7	lee, robert j.	15	837	64
8	tan, weihong	15	2053	129
9	zhang, wei	15	155	11
10	gao, feng	14	591	25
10	xia, guohua	14	334	25

### 3.5 Keyword co-occurrence, clustering, and development

**FIGURE 6:**
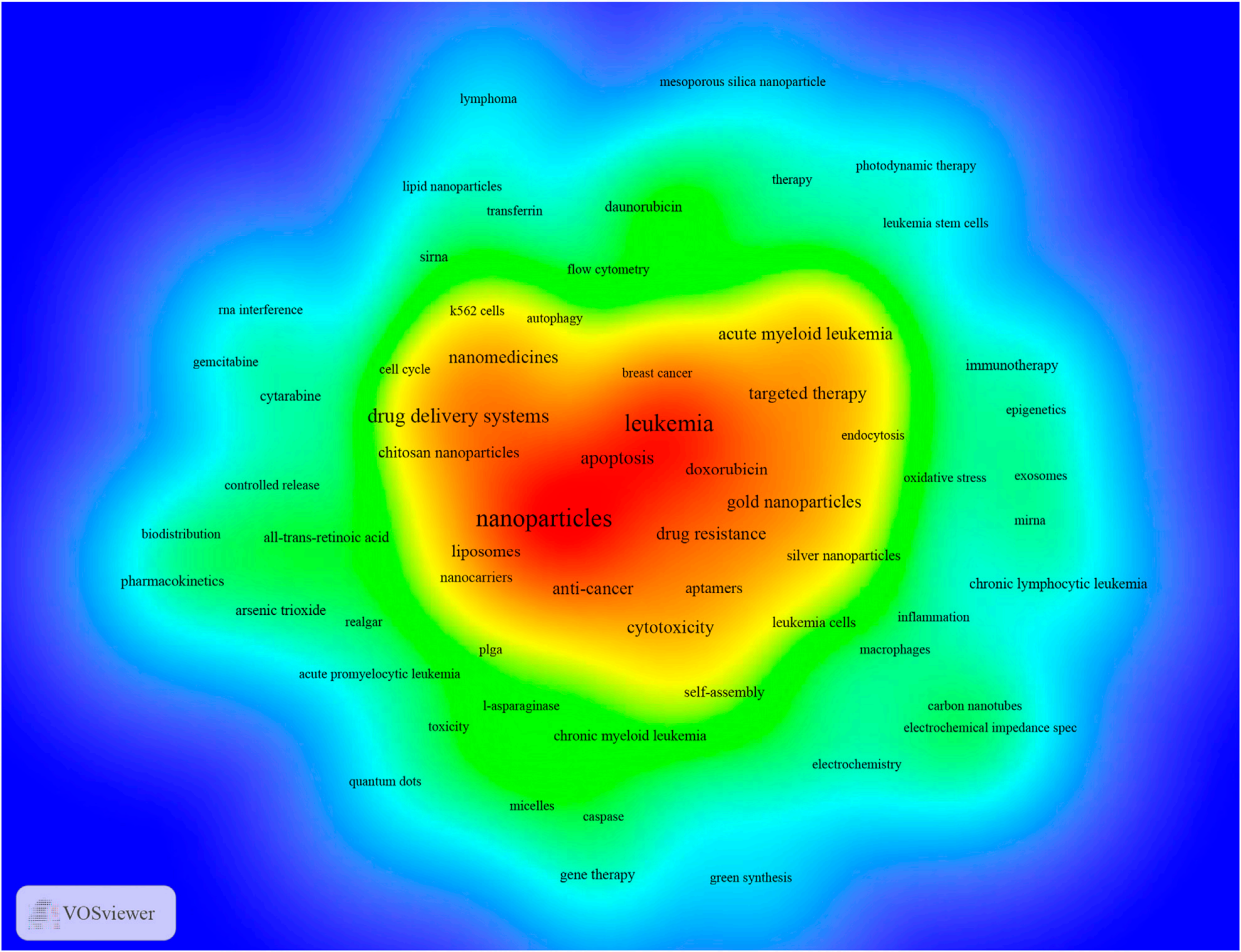
The density map of keywords in Nanoparticles-leukemia research (T≥9). Notes: The word’s size, round, and red opacity positively relate to the co-occurrence frequency.

**FIGURE 7:**
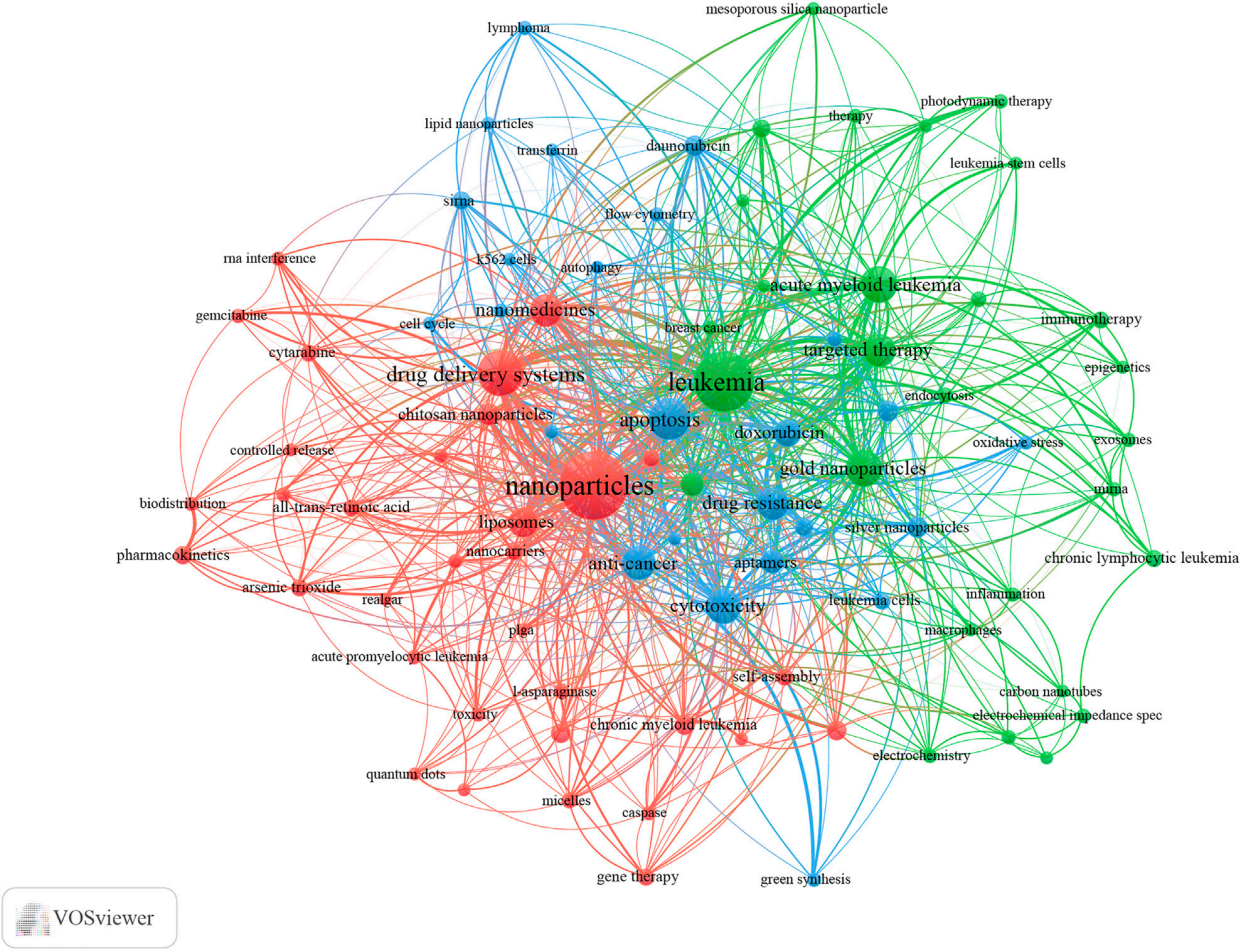
Keyword co-occurrence network and clusters in Nanoparticles-leukemia research (Notes: The size of the node and word reflects the co-occurrence frequencies, the link indicates the co-occurrence relationship, and the same color of the node represents the same cluster.

VOSviewer was utilized to present keyword co-occurrence (Table 5; Figures 6, 7) and cluster analyses (Figure 6). An Overall 4,124 keywords were abstracted, of which 90 occurred over nine times. The keyword density map (Figure 6) can find high-frequency co-occurrence entries and unveil hotspots in specific academic fields. As presented in Table 5 and Figure 6, “drug delivery systems” is a critical term, appearing 128 times in total (5.58%), followed by apoptosis, AML, gold nanoparticles, cytotoxicity, anti-cancer, targeted therapy, and drug resistance.

**TABLE 5:** The top 20 keywords of Nanoparticles-leukemia-related research.

RANK	Keyword	Occurrences	N(%)	RANK	Keyword	Occurrences	N(%)
1	nanoparticles	288	12.559965111208	11	nanomedicines	64	2.79110335804623
2	leukemia	226	9.85608373310074	12	liposomes	54	2.3549934583515
3	drug delivery systems	128	5.58220671609246	13	doxorubicin	47	2.0497165285652
4	apoptosis	107	4.66637592673354	14	aptamers	34	1.48277365896206
5	acute myeloid leukemia	85	3.70693414740515	15	magnetic nanoparticles	32	1.39555167902311
6	gold nanoparticles	81	3.53249018752726	16	chitosan nanoparticles	29	1.2647187091147
7	cytotoxicity	78	3.40165721761884	17	silver nanoparticles	28	1.22110771914522
8	anti-cancer	71	3.09638028783253	18	daunorubicin	27	1.17749672917575
9	targeted therapy	66	2.87832533798517	19	reactive oxygen species	26	1.13388573920628
10	drug resistance	64	2.79110335804623	20	chronic myeloid leukemia	24	1.04666375926734

Cluster analysis can reveal the knowledge architecture of a research field (Karwath et al., 2021). The net was separated into three clusters according to the link strength of keyword co-occurrence (Figure 7). The keywords in each cluster were highly homogeneous. Cluster 1 (red) was the most significant cluster with 33 co-occurrence keywords, including nanoparticles, drug delivery systems, nanomedicines, liposomes, chitosan nanoparticles, and nanotechnology. The keywords of Cluster 1 were mainly related to nanoparticles used in the diagnosis and treatment of leukemia. Cluster 2 (green) was primarily associated with the type and treatment of leukemia and included 35 keywords, such as leukemia, acute myeloid leukemia, gold nanoparticles, targeted therapy, magnetic nanoparticles, chemotherapy, immunotherapy, and chronic lymphocytic leukemia. Cluster 3 (blue) focused on Specific molecular mechanisms and existing problems in the application of nanoparticles in leukemia and contained 25 keywords, including apoptosis, cytotoxicity, anti-cancer, drug resistance, doxorubicin, aptamers, silver nanoparticles, daunorubicin, reactive oxygen species, and siRNA.

**FIGURE 8:**
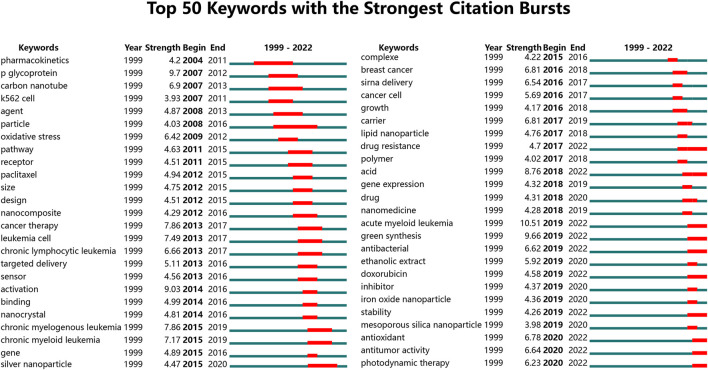
Top 50 keywords with the most vigorous citation burst r (sorted by the strength). Notes: The Blue bars mean the published reference; the red bars mean citation burstiness.

The keyword with the most powerful citation burst was developed by CiteSpace and could present the citation status of high-frequency keywords. Keywords are sorted by citation strength, with dark blue representing how long the keyword has existed and red representing the time it has been cited in a burst. High-frequency keywords (Top 50) are presented in Figure 8. As per the results, the drug resistance with high citation strength (4.7) has been in a state of citation burst in recent years and may continue to become a research hotspot in the future.

### 3.6 Co-cited reference and reference burst

**FIGURE 9:**
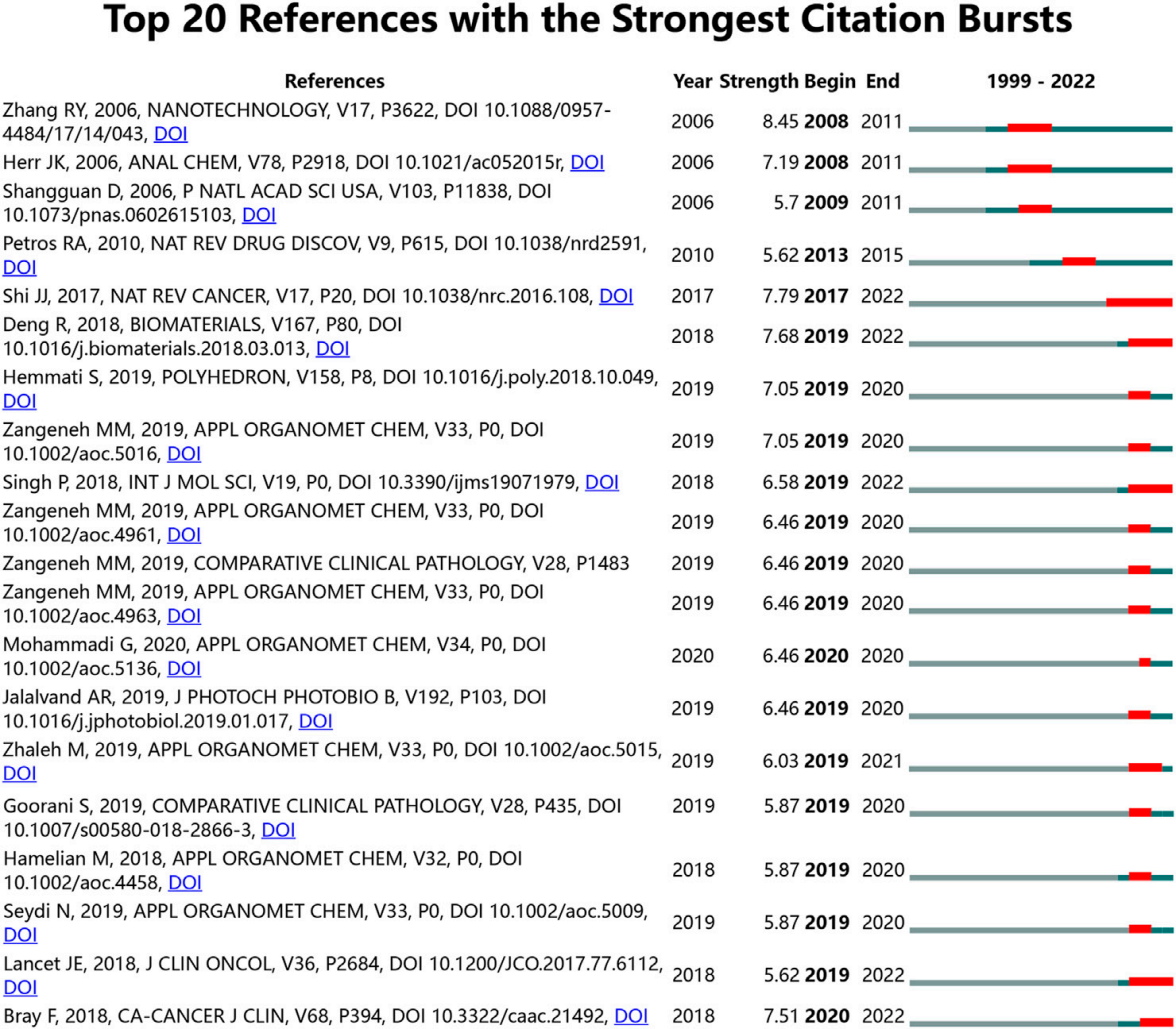
Top 20 references with the most powerful citation bursts (sorted by the strength). Notes: The Blue bars mean the published reference; the red bars mean citation burstiness.

Our team used CiteSpace to identify the co-cited references (Table 6). results show that the top 10 co-cited references were co-cited ≥ 29 times, three of which were co-cited more than 40 times. The most frequently co-cited reference is a paper written by Peer, Dan, et al. published in NATURE NANOTECHNOLOGY in 2007, entitled “Nanocarriers as an emerging platform for cancer therapy” (Peer et al., 2007), followed by an article entitled “Aptamers evolved from live cells as effective molecular probes for cancer study” (Shangguan et al., 2006). Reference with citation burst is the references often cited over time. In CiteSpace, our team set the burst duration to ≥ 2 years. We identified 99 references with the total citations and selected the top 20 references as the object of analysis. Figure 9 shows that the top 20 burst references were published after 1999 and had high citation outbreaks in 2017–2022. Notably, by 2022, 8 references (32%) were in a state of citation burst. The most substantial citation burst reference was “Synergistic enhancement effect of magnetic nanoparticles on anti-cancer drug accumulation in cancer cells.” Zhang, Renyun, and others have maintained a high citation rate since their publication in NANOTECHNOLOGY (Zhang et al., 2006).

**TABLE 6:** Top 10 co-cited references for Nanoparticles-leukemia-related research.

RANK	ID	Title	Journal	Co-citation	Year	type of article
1	Peer, Dan	Nanocarriers as an emerging platform for cancer therapy	NATURE NANOTECHNOLOGY	54	2007	review
2	Shangguan, Dihua	Aptamers evolved from live cells as effective molecular probes for cancer study	PNAS	48	2006	Article
3	Davis, Mark E.	Nanoparticle therapeutics: an emerging treatment modality for cancer	NATURE REVIEWS DRUG DISCOVERY	41	2008	review
4	Brigger, I	Nanoparticles in cancer therapy and diagnosis	ADVANCED DRUG DELIVERY REVIEWS	38	2002	review
5	Petros, Robby A	Strategies in the design of nanoparticles for therapeutic applications	NATURE REVIEWS DRUG DISCOVERY	30	2010	review
6	Connor, EE	Gold nanoparticles are taken up by human cells but do not cause acute cytotoxicity	SMALL	29	2005	Article
7	Huang, Xiaomeng	Targeted Delivery of microRNA-29b by Transferrin-Conjugated Anionic Lipopolyplex Nanoparticles:A Novel Therapeutic Strategy in Acute Myeloid Leukemia	CLINICAL CANCER RESEARCH	29	2013	Article
8	Blanco, Elvin	Principles of nanoparticle design for overcoming biological barriers to drug delivery	NATURE BIOTECHNOLOGY	29	2015	Article
9	Yechezkel, Barenholz	Doxil®--the first FDA-approved nano-drug: lessons learned	JOURNAL OF CONTROLLED RELEASE	24	2012	review
10	Jinjun Shi	Cancer nanomedicine: progress, challenges and opportunities	NATURE REVIEWS CANCER	23	2017	review

### 3.7 Analysis of journal, countries, and keywords of the top 100 most-cited articles

**FIGURE 10:**
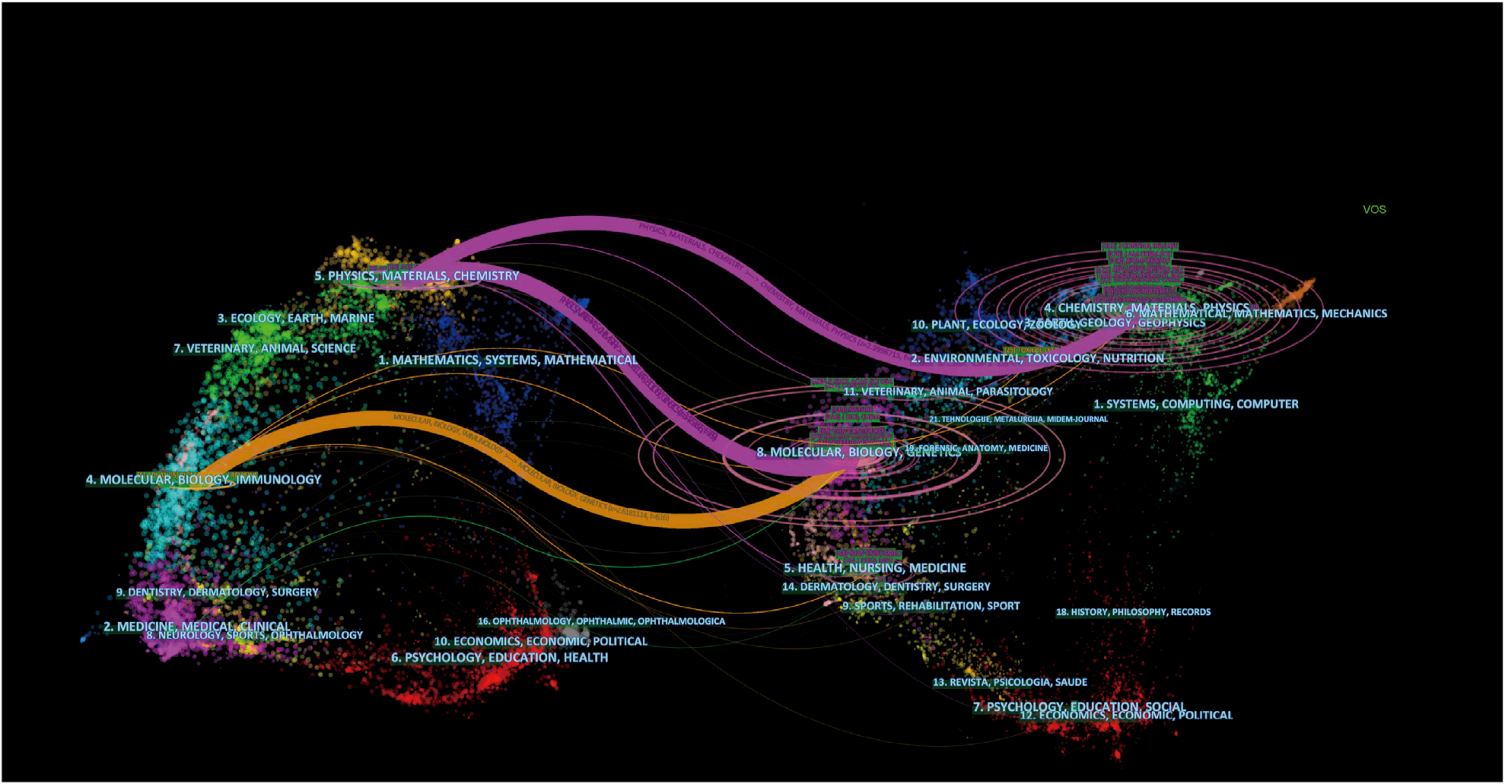
The dual-map overlay of journals related to the top 100 most cited references in Nanoparticles-leukemia research. Notes: The citing journals were on the left, the cited journals were on the right, and the colored path represents the citation relationship.

The top 100 most-cited articles were defined as cited articles with a high correlation with Nanoparticles-leukemia. We analyzed the journals and co-cited journals of the top 100 most-cited articles. (Table 7, Table 8, and Figure 10). Fifteen journals have published more than two articles, of which ACS Nano (*n* = 10), ANALYTICAL CHEMISTRY (*n* = 8), and CLINICAL CANCER RESEARCH (*n* = 5) rank top 3—followed by FRONTIERS IN BIOENGINEERING AND BIOTECHNOLOGY (*n* = 4).

**TABLE 7:** The top 10 journals of top100 cited articles research.

RANK	Journal	N	IF(2020)	JCR division	Country
1	ACS NANO	10	18.027	Q1	United States
2	ANALYTICAL CHEMISTRY	8	8.008	Q1	United States
3	ADVANCED DRUG DELIVERY REVIEWS	5	17.873	Q1	NETHERLANDS
3	BIOMATERIALS	5	15.304	Q1	NETHERLANDS
4	FRONTIERS IN BIOENGINEERING AND BIOTECHNOLOGY	4	6.064	Q1	NETHERLANDS
5	BIOSENSORS BIOELECTRONICS	3	12.545	Q1	United Kingdom
5	SMALL	3	15.153	Q1	GERMANY
6	INTERNATIONAL JOURNAL OF NANOMEDICINE	2	7.033	Q1	New Zealand
6	JOURNAL OF THE AMERICAN CHEMICAL SOCIETY	2	16.383	Q1	United States
6	MOLECULAR THERAPY	2	12.91	Q1	United States
	NANO LETTERS	2	12.262	Q1	United States
	NANOMEDICINE	2	6.096	Q1	United Kingdom
	NANOMEDICINE NANOTECHNOLOGY BIOLOGY AND MEDICINE	2	6.458	Q2	United States
	PNAS	2	12.779	Q1	United States
	SCIENTIFIC REPORTS	2	4.996	Q2	United Kingdom

**TABLE 8:** The top co-cited 20 journals of top100 cited articles research.

RANK	Co-cited journal	Citations	IF(2021)	JCR division	Country
1	PNAS	233	12.779	Q1	United States
2	CANCER RESEARCH	217	13.312	Q1	United States
3	JOURNAL OF CONTROLLED RELEASE	202	11.467	Q1	NETHERLANDS
4	JOURNAL OF THE AMERICAN CHEMICAL SOCIETY	176	16.383	Q1	United States
5	BIOMATERIALS	156	15.304	Q1	NETHERLANDS
6	ACS NANO	143	18.027	Q1	United States
7	BLOOD	141	25.476	Q1	United States
8	SCIENCE	140	63.714	Q1	United States
9	NATURE	139	69.504	Q1	United Kingdom
10	ANALYTICAL CHEMISTRY	131	8.008	Q1	United States
11	ADVANCED DRUG DELIVERY REVIEWS	125	17.873	Q1	NETHERLANDS
12	JOURNAL OF BIOLOGICAL CHEMISTRY	116	5.486	Q2	AUSTRALIA
13	ANGEWANDTE CHEMIE-INTERNATIONAL EDITION	108	16.823	Q1	GERMANY
14	NANO LETTERS	102	12.262	Q1	United States
15	BIOCONJUGATE CHEMISTRY	92	6.069	Q1	United States
16	NATURE BIOTECHNOLOGY	74	68.164	Q1	United States
17	SMALL	70	15.153	Q1	GERMANY
18	CLINICAL CANCER RESEARCH	69	13.801	Q1	United States
19	LANGMUIR	65	4.331	Q2	United States
20	NATURE REVIEWS CANCER	57	69.8	Q1	United Kingdom
21	MOLECULAR PHARMACEUTICS	56	5.364	Q1	United States
22	NATURE NANOTECHNOLOGY	56	40.523	Q1	United Kingdom
23	NATURE REVIEWS DRUG DISCOVERY	54	112.288	Q1	United Kingdom
24	ADVANCED MATERIALS	52	32.086	Q1	GERMANY
25	JOURNAL OF CLINICAL ONCOLOGY	52	50.717	Q1	United States
26	CELL	48	66.85	Q1	United States
27	NEW ENGLAND JOURNAL OF MEDICINE	48	176.079	Q1	United States
28	INTERNATIONAL JOURNAL OF CANCER	43	7.033	Q2	NEW ZEALAND
29	NANOMEDICINE	42	6.096	Q1	United Kingdom
30	FRONTIERS IN BIOENGINEERING AND BIOTECHNOLOGY	40	6.064	Q1	NETHERLANDS

In the co-cited journals, PROCEEDINGS OF THE NATIONAL ACADEMY OF SCIENCES OF THE UNITED STATES OF AMERICA (*n* = 233) was the most common, followed by CANCER RESEARCH (*n* = 217), JOURNAL OF CONTROLLED RELEASE (*n* = 202), and JOURNAL OF THE AMERICAN CHEMICAL SOCIETY (*n* = 176). Interestingly, in the top 100 most-cited articles field, FRONTIERS IN BIOENGINEERING AND BIOTECHNOLOGY is not only a high publication volume journal but also a high co-citation journal. Roughly consistent with the overall field analysis, journal analysis of the top 100 most-cited articles identified three primary orange citation paths, which suggests that research published in the Physics/Materials/chemistry and Molecular/Biology/Immunology journal is predominantly cited by a study published in the Molecular/Biology/Genetics and Chemistry/Materials/Physics journal.

**FIGURE 11:**
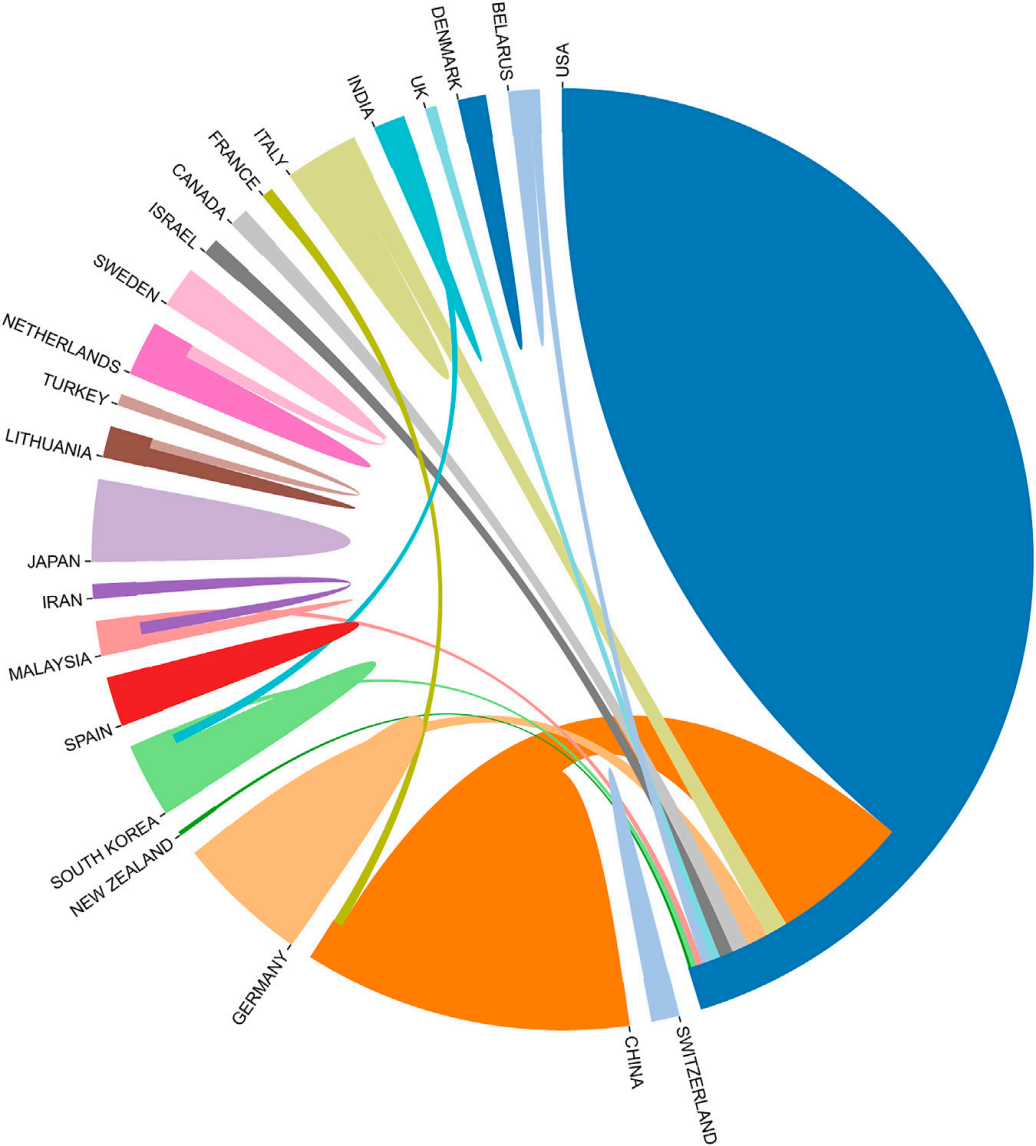
Country-to-country relations of Top 100 Most-cited Articles.

**FIGURE 12:**
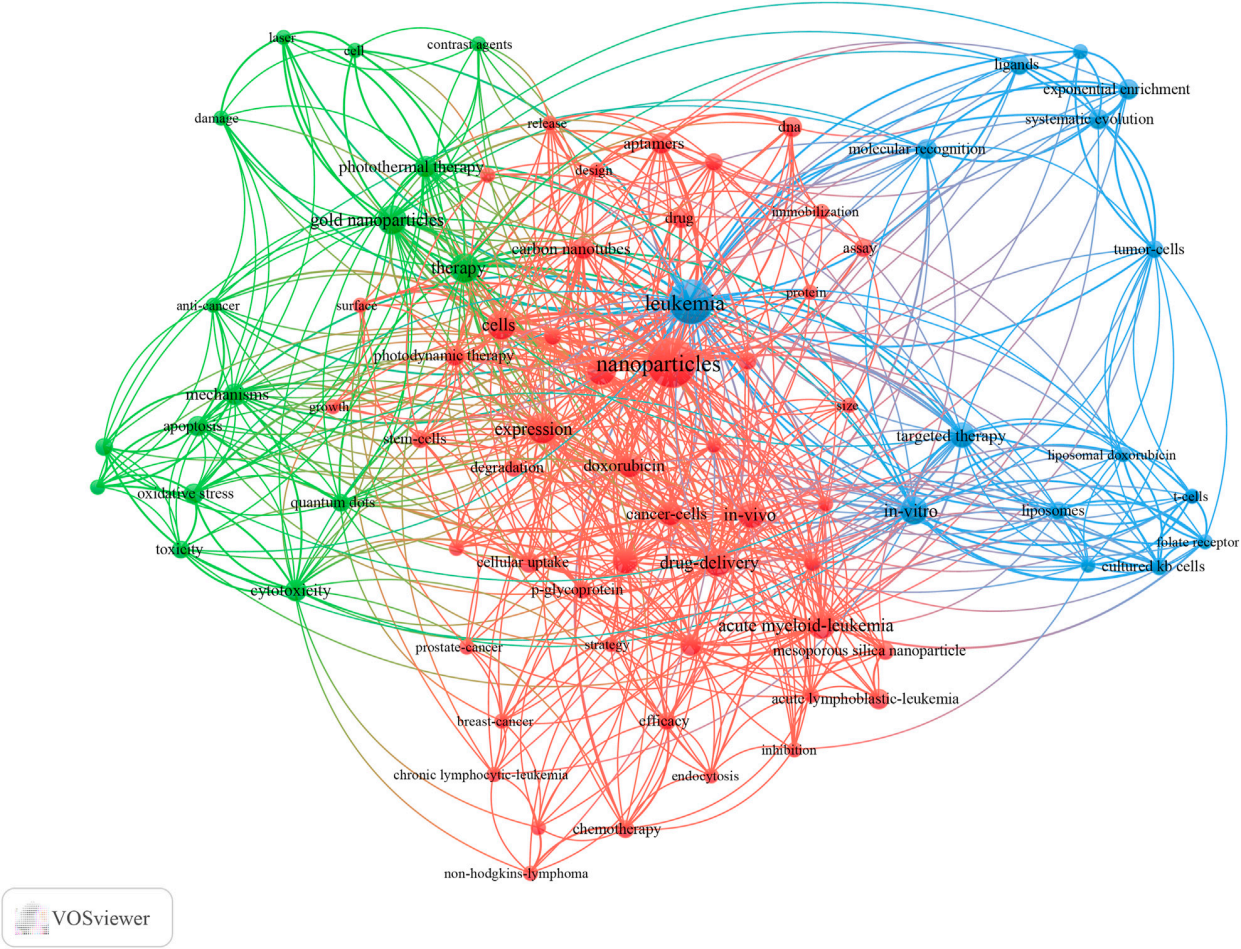
Keyword co-occurrence network and clusters in Nanoparticles-leukemia research. Notes: The size of the node and word reflects the co-occurrence frequencies, the link indicates the co-occurrence relationship, and the same color of the node represents the same cluster.

We also analyzed the countries with the top 100 most-cited articles (Figure 11). United States (*n* = 54) was the nation with the most papers, followed by China (*n* = 29). China has frequent academic exchanges with the United States, while the United States has ties with ten countries, including CHINA, JAPAN, and GERMANY. To further analyze the development content and trend of the Nanoparticles-leukemia field, we analyzed the keywords of the top 100 articles. The keyword of the top 20 (Table 9) coincided with the top 20 (Table5) in the Nanoparticles-leukemia field, such as targeted therapy, nanoparticles, leukemia, aptamers, drug-resistance, and drug delivery systems, doxorubicin, cytotoxicity, apoptosis, and acute myeloid leukemia. This shows that the primary research content of Nanoparticles-leukemia is related to the above keywords. The net was separated into three clusters according to the link strength of keyword co-occurrence (Figure 12). Cluster 1 (red) was the most significant cluster with 49 co-occurrence keywords, including nanoparticles, acute myeloid-leukemia, drug delivery system, doxorubicin, and aptamers. The theme of Cluster 1 is highly correlated with the design strategy of the nano drug delivery system. Cluster 2 (green) was primarily associated with the specific mechanism and possible hindrance of the anti-cancer effect of nano-drugs and included 16 terms, such as anti-cancer, apoptosis, cytotoxicity, damage, drug resistance, therapy, and toxicity. Cluster 3 (blue) focuses on immunotherapy and targeted therapy related to the nano-drug delivery system and contains 15 terms, such as folate receptor, gene therapy, ligands, liposomal doxorubicin, liposomes, molecular recognition, t-cells, targeted therapy, and tumor-cells.

**TABLE 9:** The top 20 keywords of top100 cited articles research.

RANK	Keyword	Occurrences	N(%)	RANK	Keyword	Occurrences	N(%)
1	nanoparticles	32	7.35632183908046	11	in-vivo	10	2.29885057471264
2	leukemia	25	5.74712643678161	12	targeted therapy	8	1.83908045977011
3	expression	12	2.75862068965517	13	cancer-cells	7	1.60919540229885
4	cells	11	2.52873563218391	14	doxorubicin	7	1.60919540229885
5	delivery	11	2.52873563218391	15	aptamers	6	1.37931034482759
6	gold nanoparticles	11	2.52873563218391	16	carbon nanotubes	6	1.37931034482759
7	therapy	11	2.52873563218391	17	cytotoxicity	6	1.37931034482759
8	acute myeloid-leukemia	10	2.29885057471264	18	mechanisms	6	1.37931034482759
9	drug-delivery	10	2.29885057471264	19	drug-resistance	6	1.37931034482759
10	in-vitro	10	2.29885057471264	20	photothermal therapy	6	1.37931034482759

## 4 Discussion

### 4.1 General information

According to data from the WoSCC database, as of 2 August 2022, 8,741 authors from 2,174 institutions in 80 countries have published 1825 studies on Nanoparticles-leukemia in 476 academic journals.

The annual output change and cited frequency are essential indicators of the development trends in this field. In the included literature, Luisa T ondelli et al. constructed polymer nanospheres that can increase leukemia cells’ uptake of antisense oligonucleotides (Tondelli et al., 1999). Since then, related articles in the field of Nanoparticles-leukemia have shown an upward trend (Figure 2). Nanoparticles-leukemia-related articles can be separated into 3 phases, namely “budding,” “steady growth,” and “fast developmental process.” “Budding” (1999–2009): The concept of the effect of Nanoparticles on leukemia research is beginning to appear. In these 11 years, no more than 50 articles were published each year, and the annual citation frequency was less than 1000. “Steady growth” (2010–2016): Nanoparticles-leukemia received more attention from scientists, and the output every year increased stably. “Fast developmental process” (2017 to present): At this stage, not only did the number of articles gradually increase steadily but also the citation frequency showed a turning rise, indicating that Nanoparticles-leukemia research has been paid more attention increasingly by researchers and developed rapidly. Furthermore, the growth trends in this field are promising.

The analysis of journals and co-cited journals (Tables 1, 2) revealed that the INTERNATIONAL JOURNAL OF NANOMEDICINE published the most significant number of studies and obtained more co-cited references in the Nanoparticles-leukemia field. This means that the journal has a strong influence in the field of nano-leukemia. The dual-map overlay of journals represents their theme distributional status (Figure 3). This overlay exhibited four central citation paths from Molecular/Biology/Immunology and Physics/Materials/chemistry journals to Molecular/Biology/Genetic and Chemistry/Materials/Physics co-cited journals. Concurrently, journals at the Q1 JCR division with high IF took up half of the top 10 journals (75%) and co-cited journals (100%), indicating that these journals publish studies on and are vital for Nanoparticles-leukemia associated research. The analysis of countries/institutions related to Nanoparticles-leukemia studies shows (Table 3; Figure 4) that China, the United States, and India are the top three producers.

However, the United States, Germany, France, and Canada could potentially induce revolutionary breakthroughs. Italy, the United States, Germany, and South Korea were the first nations to carry out Nanoparticles-leukemia-related studies, followed by China and France. This shows that the United States has always been fruitful and influential in studying Nanoparticles-leukemia. Notably, China started relatively late but has become one of the most productive contributors in recent years. This may be related to the increased attention due to the high incidence and mortality of Leukemia in China.

Furthermore, cooperation between diverse nations, particularly America, is very active, revealing that research on Nanoparticles-leukemia has aroused widespread concern worldwide, and the United States is the primary cooperation center. The top 11 organizations come from three nations: 8 out of 11 are from China, 2 out of 11 are from the United States, and one is from Iran. Moreover, our team discovered fruitful collaboration among Southeast Univ, Chinese Acad Sci, Nanjing Univ, and other organizations, which has contributed remarkably to the field of Nanoparticles-leukemia.

Highlighting the contributions of active scholars, such as those who co-appear or co-cite articles in a particular field, can assist researchers in moving forward along this path and offer more directions and guidance. Here (Table 4; Figures 4, 5), wang and Xue mei published the most papers. Additionally, maps of researchers and co-cited researchers provide data regarding the underlying cooperators and powerful academic teams. Scholars have actively collaborated within and between organizations in Nanoparticles-leukemia, particularly among researchers. Overall, 20 scholars from 4 organizations published a critical review titled “Targeted Delivery of microRNA-29b by Transferrin-Conjugated Anionic Lipopolyplex Nanoparticles: A Novel Therapeutic Strategy in Acute Myeloid Leukemia” (Huang et al., 2013). This suggests that these powerful groups might be the underlying cooperators of scholars.

### 4.2 Knowledge base

The co-cited references have been cited together in other articles. Nevertheless, the knowledge base is a pool of co-cited references cited by relevant academic teams, which is not entirely equal to frequently cited references. In the bibliometric analyses, In the Nanoparticles-leukemia field, there are many overlapping parts in the top 10 high co-cited articles and the top 20 citation burst articles (Table 6; Figure 9). This shows that the knowledge base constructed in this study has a substantial reference value.

In 2007, NATURE NANOTECHNOLOGY published the most co-cited Nanoparticles-leukemia studies by Peer, Dan, et al. (*n* = 54) (Peer et al., 2007). This article reviews the different mechanisms of targeted delivery of nanomedicines to tumors and reports the research progress of nano-based platforms for treating leukemia. Shangguan, Dihua et al. wrote the second co-citation research on PROCEEDINGS OF THE NATIONAL ACADEMY OF SCIENCES OF THE UNITED STATES OF AMERICA (Shangguan et al., 2006). This study developed a strategy by which a set of aptamers for specific recognition of leukemic cells could be generated, and it is also a Citation Burst article (Strength = 5.7, duration = 2009–2011). Davis, Mark E. et al. (2008) published the third co-cited article in the NATURE REVIEWS DRUG DISCOVERY. (Davis et al., 2008). Unlike the most co-cited reference, this review mainly summarized the application of nanoparticles in preclinical and clinical studies, focusing on the application of nanoparticles in leukemia. The fourth co-cited paper was published in the Nanoparticles in cancer therapy and diagnosis by Brigger et al. (2002). In 2002. This review provides an update on using conventional or long-cycle nanoparticles to target tumors. In 2015, Petros, Robby A et al. reported the fifth co-cited study in the NATURE REVIEWS DRUG DISCOVERY. (Petros and DeSimone, 2010); this review highlights recent advances that are important for the rational design of such nanoparticles and discusses the challenges to realizing their potential and it is also a Citation Burst article (Strength = 5.62, duration = 2013–2015). In the sixth article, published by Connor et al. (2005). In 2005, gold nanoparticles are studied in this paper. Although gold nanoparticles can be ingested by leukemia cells and cause cytotoxicity, they can be non-toxic by surface modification. In 2013, CLINICAL CANCER RESEARCH published the seventh co-citation study by Huang et al. (2013). The research team mainly used transferrin-conjugated anionic lipid polymers to construct composite nano-drugs for the treatment of leukemia, which carried a mimic of microRNA-29b. The eighth co-cited article was written by Blanco, Elvin, et al. in NATURE BIOTECHNOLOGY, 2015. (Blanco et al., 2015). This paper mainly reviews the physical obstacles in nanoparticle transport and the solutions to overcome the biological obstacles. It is also explained that the future research focus of nanoparticles in leukemia is to use a nanoparticles-based platform for gene delivery. Notably, it is a Citation Burst article (Strength = 5.33, duration = 2018–2019). In 2012, the JOURNAL OF CONTROLLED RELEASE published the ninth co-citation research completed by Yechezkel, Barenholz. This review summarizes the history and scientific viewpoints of the development of Doxil, as well as the experiences and lessons learned from its development, and discusses its application in leukemia (Zucker et al., 2012). In 2017, the 10th co-cited paper in the NATURE REVIEWS CANCER by Jinjun Shi and others. This review summarizes the historical schedule of significant developments in cancer nanomedicine, predicts the potential markers of the EPR effect and nano therapy effect, and summarizes the clinical application of nanomedicine in leukemia (Deng et al., 2018).

Overall, the top 10 co-cited papers highlighted reviews (six reviews completed in a different year), different nanoparticles used in the treatment of leukemia (Small-Molecule Drugs, Small-Molecule Drugs, Nanoparticles for Gene Therapy, Nanoparticles for Combined Therapy, and Nanoparticles for Photodynamic Therapy), the status of leukemia treatment (Chemotherapy, Radiation Therapy, Radiation Therapy, Targeted Therapy) and Obstacles of nano-drugs in the treatment of leukemia (safety, targeting, inadequate drug release response, drug resistance).

### 4.3 Hot topic development, knowledge structure, and emerging topics

In bibliometric analysis, keyword/term co-occurrence (Table 5; Figure 6, and Figure 7) can indicate a hot spot in an academic field, and the keyword citation burst (Figure 8) can display the evolutionary process of novel hot spots. High-frequency keywords (Table 5, 9 and Figure 7) include targeted therapy, nanoparticles, leukemia, aptamers, drug resistance, drug delivery systems, doxorubicin, cytotoxicity, apoptosis, and acute myeloid leukemia, which are considered the focus of Nanoparticles-leukemia research. With time, new topics continue to emerge (Figure 8). During the nascent stage (1999–2009), emerging keywords include carbon nanotube, oxidative stress, P-glycoprotein, and so on. This is also in line with the actual situation. At this stage, researchers tried to clarify the pathogenesis of leukemia. In the steady growth phase (2010–2016), These keywords are mainly related to the Researchers who have done much research on nano-drugs in the treatment of cancer, so keywords related to anti-cancer nano-drugs have emerged (cancer therapy, targeted delivery, silver nanoparticle, paclitaxel, and nanocomposite). In the rapid developmental phase (2017–present), with the clinical application of nano-drugs (lipid nanoparticle, liposome, polymer), clinical-related problems (drug resistance, stability) and more nano-synthesis strategies have emerged (green synthesis). Unfortunately, although many studies have focused on solving the problem of drug resistance in leukemia, there is still no good solution.

In addition, keyword clusters can indicate knowledge of the inner architecture and unveil academic frontiers of these disciplines. Cluster analyses show three primary clusters in the field of Nanoparticles-leukemia (Figures 7, 12): nanoparticles for the diagnosis and treatment of leukemia, related to the type and treatment of leukemia, the specific molecular mechanism, and existing problems of the application of nanoparticles in leukemia. As shown earlier, anti-cancer drug resistance remains a significant obstacle to leukemia treatment. It is urgent to explore the mechanisms of drug resistance (multi-drug efflux pumps, drug metabolic enzymes, secondary mutations, remodeling of bone marrow microenvironment, metabolic changes, abnormal activation of signal pathways) and develop targeted and drug-resistant nano-drugs.

Studies presenting strongly-cited breakthroughs (Figure 9) can also characterize new topics in a field. The article with the most vigorous citation burst intensity (strength = 8.45) is a monograph published by Chen Baoan from Southeast University in NANOTECHNOLOGY (Zhang et al., 2006). This article found that magnetic nanoparticles can increase the accumulation of chemotherapeutic drugs in leukemia-resistant cell lines, resulting in different nanoparticles. Different properties of the particles can have wildly different effects on cells. At the same time, to this day, 7 of the 25 highly cited articles are still in a state of the continuous outbreak. These seven references reflect the most recent Nanoparticles-leukemia themes and are, therefore, worthy of further discussion. We rank the eight articles according to the strength of citation bursts, and the first continuous citation of outbreaks paper is also a high Co-cited paper (strength = 7.79), which shows that this paper has an indelible contribution in the field of nano-leukemia (Shi et al., 2017). The second continuous citation of outbreaks paper (strength = 7.68) was published on BIOMATERIALS by Professor Yan Fei of Jilin University, which designed functionalized gold nanoparticles that can treat AML by affecting DNA methylation (Deng et al., 2018). The third continuous citation of outbreaks paper (strength = 7.51) is from the American Cancer Society’s Scientific Vice Presiden Ahmedin Jemal, published at CA-A CANCER JOURNAL FOR CLINICIANS. This paper provides a comprehensive overview of global cancer and focuses on the global situation of leukemia (Bray et al., 2018). The fourth-ranked literature (strength = 6.58) is a blockbuster review of INTERNATIONAL JOURNAL OF MOLECULAR SCIENCES published by Ivan Mijakovic of Chalmers University of Technology, which comprehensively summarizes the use of gold nanoparticles in human cancer (Singh et al., 2018).Interestingly, the paper also cited the sixth high co-cited literature and agreed that gold nanoparticles of a particular size might not be toxic to leukemic cells. The fifth-ranked literature (strength = 5.62) is a clinical study published by Ivan Mijakovic and JOURNAL OF CLINICAL ONCOLOGY, which focuses on CPX-351 (a dual liposome encapsulation of cytarabine and daunorubicin) (Lancet et al., 2018). Compared with traditional 7 + 3, CPX-351 can significantly prolong the survival time of leukemia patients with the same safety. This shows that nano-drugs can not only play an anti-cancer role in the theory but also improve patients’ quality of life.

To precisely analyze Nanoparticles-leukemia research-related content, we analyzed journals, co-cited journals, and national and keyword analyses of the top most cited 100 articles (Parmar et al., 2019; Mainwaring et al., 2020). The study of journals and co-cited journals (Tables 7, 8) demonstrated that ACS NANO, ANALYTICAL CHEMISTRY, and BIOMATERIALS published the highest number of articles on Nanoparticles-leukemia but also acquired the most co-cited references. The contribution of FRONTIERS IN BIOENGINEERING AND BIOTECHNOLOGY in Nanoparticles-leukemia research is also noteworthy. In the field of top100, the journal is not only a high-output journal and a highly co-cited journal but is also published as a highly cited journal in this field. Larissa Pereira Brumano summarized the role of L-asparaginase in treating acute lymphoblastic leukemia and the existing methods to improve the biocompatibility and therapeutic efficiency of L-asparaginase (Brumano et al., 2019). This study will facilitate follow-up scholars to gain an in-depth understanding of the mechanisms involved in ASNase treatment to develop new and effective strategies to improve this biopharmaceutical.

The dual-map overlay analyses of the top 100 most-cited articles as well as all articles in the field (Figure 10) show 3 of the primary citation paths from Physics/Materials/chemistry and Molecular/Biology/Immunology co-cited journals to Molecular/Biology/Genetics and Chemistry/Materials/Physics journals, which indicates that Nanoparticles-leukemia studies not only focused on molecular biology but also closely related to the field of materials and physics. The nation-wise analysis findings of the top 100 most-cited papers are consistent with those of all articles in the field, showing that the United States, CHINA, and GERMANY are the largest producers. Additionally, cooperation between the United States and other countries is active, indicating that Nanoparticles-leukemia-related research has attracted worldwide attention.

### 4.4 The possible focus of future research

The existing treatment methods for leukemia mainly include chemotherapy, radiotherapy, etc. However, the emergence of drug resistance significantly hinders the efficacy of chemotherapy and leads to a poor prognosis, and the side effects caused by poor targeting also make patients miserable. How to solve the chemotherapy resistance, improve the prognosis and improve the quality of life of patients has become a thorny problem. With the rise of nanomedicine and targeted therapy, future researchers can explore the mechanism of drug resistance and active targeting and synthesize nano-drugs that have both targeted therapy and chemotherapy resistance according to the mechanism, which may be the dawn of the solution of leukemia.

## 5 Limitation

The present study had some inherent flaws in bibliometrics. First, data were acquired from the WoSCC database, excluding some research not in WoSCC. Nevertheless, WoSCC is the most frequently used scientific econometric research database; data from WoSCC can cover the majority of information to a certain extent. Second, all data were acquired *via* bibliometric tools based on machine learning and natural language processing, which may cause biases in other bibliometric studies. Third, in the included article, part of the review also describes diseases other than leukemia, which may lead to analysis deviation. Nevertheless, compared to the most recent conventional reviews, the results herein are consistent and provide scholars with more objective data and insights.

## 6 Conclusion

In summary, research on Nanoparticles-leukemia is fast-developing, and global collaboration is active, wherein America and China is the primary collaboration center. Currently, the research predominantly highlights the fields of molecular biology, Immunology, physics, materials, and chemistry. The three primary aspects of Nanoparticles-leukemia-related studies include nanoparticles for the diagnosis and treatment of leukemia, the type and treatment of leukemia, the specific molecular mechanism, and existing problems of the application of nanoparticles in leukemia. Future research may be to synthesize nanoparticles with both targeting and reversible drug resistance. Our study is the first to research Nanoparticles-leukemia-associated articles using bibliometrics and knowledge graph systems. INTERNATIONAL JOURNAL OF NANOMEDICINE is the journal with the highest output. The contribution of FRONTIERS IN BIOENGINEERING AND BIOTECHNOLOGY is also noteworthy.In contrast to conventional reviews, the present study offers preliminary and objective insights into Nanoparticles-leukemia studies. We believe that the results of the current report will provide valuable references for future research.

## Data Availability

The original contributions presented in the study are included in the article/supplementary material, further inquiries can be directed to the corresponding authors.
